# Advancing regenerative therapies with umbilical cord-derived mesenchymal stem cells: A review

**DOI:** 10.17305/bb.2025.13147

**Published:** 2025-10-01

**Authors:** Mohamed Hussein

**Affiliations:** 1 Department of Biomedical Sciences, Dubai Medical College for Girls, Dubai Medical University, Dubai, UAE

**Keywords:** Regenerative medicine, clinical studies, immunomodulation, anti-inflammatory therapy, tissue repair and regeneration

## Abstract

Umbilical cord-derived mesenchymal stem cells (UC-MSCs) are a clinically attractive regenerative and immunomodulatory platform that combines ethical accessibility, low immunogenicity, rapid expansion, genetic stability, and a potent paracrine secretome. This study aimed to synthesize evidence on safety, efficacy, and translational readiness by conducting a focused PubMed review (2014–2024) restricted to clinical studies and trials, using predefined inclusion and exclusion criteria and structured data extraction. Across indications, UC-MSCs show a consistent safety profile and signals of benefit mediated by tissue repair and immune regulation: in musculoskeletal disease they improve osteoarthritis pain and function and may slow osteonecrosis; in hepatology they sustain gains in decompensated cirrhosis, mitigate acute allograft rejection, and aid recovery from ischemic-type biliary lesions; as induction in renal transplantation they are feasible with early graft benefits; in type 2 diabetes responders improve glycemic control and inflammation, while maternal and obstetric factors can shape intrinsic cell properties; in neurology, studies in cerebral palsy, chronic spinal cord injury, and traumatic optic neuropathy report motor, sensory, and visual improvements; in COVID-19-related acute respiratory distress syndrome (ARDS) trials show better oxygenation, radiological recovery, quality of life, and modulation of the TNF-sTNFR2 axis; in immune-mediated and transplant settings they reduce graft-versus-host disease, with signals in systemic lupus erythematosus, refractory immune thrombocytopenia, Crohn’s fistulas, and as cotransplant support in aplastic anemia. The limitations of this study encompass small sample sizes, single-center designs, and short-duration trials. Additionally, there is significant heterogeneity concerning the source, manufacturing processes, dosage, administration routes, and endpoints. Other challenges include adherence to good manufacturing practices (GMP), issues related to potency, biobanking, logistical constraints, cost factors, and regulatory obstacles. Large multicenter randomized trials with standardized protocols and long-term follow-up, and combination strategies with biomaterials, gene engineering, and extracellular vesicle or exosome products, are needed to confirm durable benefit and enable routine clinical integration.

## Introduction

Mesenchymal stem cells (MSCs) are multipotent stromal cells characterized by their ability to self-renew and differentiate into various cell types, including adipocytes, chondrocytes, and osteoblasts. These attributes render MSCs highly valuable in regenerative medicine, tissue engineering, and immunotherapy [1]. MSCs are identified by their absence of hematopoietic markers such as CD34 and CD45, their expression of specific markers including CD73, CD90, and CD105, and their capacity to adhere to plastic surfaces in culture [2]. Beyond their differentiation capabilities, MSCs possess significant immunomodulatory effects, interacting with immune cells such as T cells, B cells, and macrophages [3].

**Figure 1.**
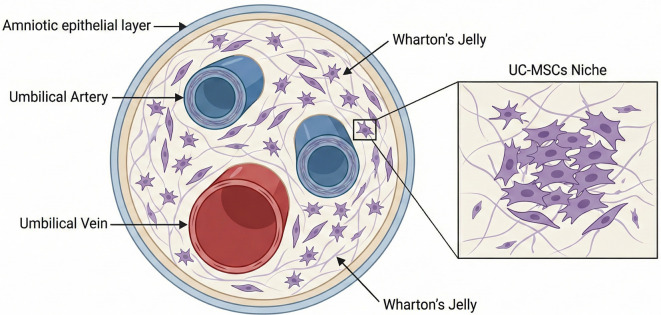
**Cross-sectional schematic of the human umbilical cord highlighting the anatomical source of Wharton’s jelly and umbilical cord-derived mesenchymal stem cells (UC-MSCs).** The illustration depicts a cross-section of the umbilical cord, highlighting two umbilical arteries and a central umbilical vein containing cord blood, all encapsulated within Wharton’s jelly. Wharton’s jelly serves as a rich extracellular matrix that supports the presence of UC-MSCs, which can be efficiently isolated for regenerative and therapeutic purposes. This schematic underscores the vascular structures and the adjacent stromal compartment, identifying them as the primary niche for UC-MSCs.

MSCs can be sourced from various tissues, each presenting unique advantages and limitations. Bone marrow is the most well-established source, known for its high differentiation potential; however, its collection is invasive, and yield diminishes with age. Adipose tissue offers a less invasive alternative with good availability, though it exhibits slightly reduced osteogenic capacity [4]. Umbilical cord tissue, particularly Wharton’s jelly, serves as an abundant and non-invasive source, although these cells may demonstrate lower self-renewal potential compared to other sources. Amniotic fluid and membranes also show promise due to their pluripotent-like properties, yet they raise ethical considerations. Additional sources, including peripheral blood, dental pulp, synovial fluid, liver, lung, and skeletal muscle, have been investigated but often suffer from low yield and limited accessibility [5] (Figure 1). Among these sources, umbilical cord-derived MSCs (UC-MSCs) are emerging as a leading candidate for clinical applications. Derived from Wharton’s jelly, cord blood, or perivascular tissue, UC-MSCs can differentiate into multiple lineages and are readily accessible from tissue typically discarded after birth. Their collection is safe, non-invasive, and ethically acceptable, making them a particularly valuable resource for advancing regenerative therapies and immune-based treatments [6, 7].

UC-MSCs are attracting increasing attention in regenerative medicine due to their combination of accessibility, safety, and biological efficacy [8]. They can be harvested easily and non-invasively since the umbilical cord is usually discarded after childbirth, thereby mitigating ethical concerns and donor risks [9]. This characteristic positions them as a widely available and ethically sound source of stem cells. A significant advantage of UC-MSCs is their “youthful” biology, as they originate from neonatal tissue. They exhibit rapid proliferation, long-term genetic stability, and resistance to cellular aging [10]. These attributes facilitate the expansion of large, clinically relevant cell populations without compromising cellular integrity. Functionally, UC-MSCs can differentiate into multiple lineages, including bone, cartilage, and adipose tissue, while secreting a diverse array of bioactive molecules that regulate immune responses and suppress inflammation [11]. Such immunomodulatory properties render them particularly promising for treating inflammatory and immune-related diseases, where traditional therapies often fall short [12].

**Figure 2.**
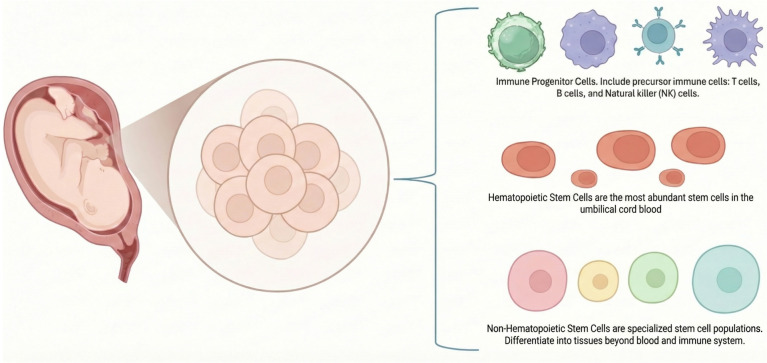
**Illustration of the umbilical cord as a rich source of diverse stem and progenitor cells with significant therapeutic potential.** Hematopoietic stem cells (HSCs) generate all types of blood cells and are extensively utilized in hematologic therapies. Immune progenitor cells, which differentiate into T cells, B cells, and natural killer (NK) cells, play a pivotal role in immune regulation and transplantation. Mesenchymal stem cells (MSCs) can differentiate into various tissues while exerting significant immunomodulatory and regenerative effects. Additionally, non-hematopoietic stem cells contribute to neural and cardiac repair. Collectively, these cell populations underscore the umbilical cord’s potential as an ethically accessible and versatile source for regenerative medicine, immunotherapy, and hematologic treatments.

Another critical advantage of UC-MSCs is their robust safety profile. Being derived from neonatal tissue, they harbor fewer accumulated genetic mutations compared to adult-derived stem cells, thereby reducing concerns regarding malignant transformation [13]. Additionally, unlike embryonic stem cells, they circumvent significant ethical controversies, as their source material would otherwise be discarded as medical waste. Collectively, UC-MSCs represent a highly reliable and versatile platform for advancing cell-based therapies. Their combination of ethical accessibility, strong biological performance, and proven immunomodulatory effects positions them as a cornerstone resource for the future of regenerative medicine and immune-targeted treatments [14] (Figure 2).

## Methods

A comprehensive literature search was conducted in PubMed to identify studies on UC-MSCs published between 2014 and 2024. The search utilized Medical Subject Headings (MeSH) and free-text terms, including “Umbilical cord-derived mesenchymal stem cells,” “UC-MSCs AND therapy,” and “UC-MSCs AND immune disorders,” employing Boolean operators and field tags ([Title/Abstract], [MeSH Terms]) to refine the results. Filters were applied to include only clinical studies and trials, with additional restrictions on disease-specific topics such as liver cirrhosis, immune disorders, COVID-19, and metabolic diseases. The selected time frame of 2014–2024 encompasses a decade of significant growth in UC-MSC research, highlighting major advances in clinical translation and the publication of large-scale trials. PubMed was chosen as the primary database due to its extensive coverage of biomedical and clinical research, particularly peer-reviewed journal articles indexed in MEDLINE, which ensures the reliability and quality of sources.

Retrieved records were imported into Excel, duplicates were removed, and titles and abstracts were screened for relevance, followed by a full-text assessment for eligible studies. Inclusion criteria consisted of clinical studies (randomized controlled trials, pilot studies, and phase I–III trials) involving UC-MSCs as a therapeutic intervention and reporting outcomes on efficacy, safety, or therapeutic potential. Exclusion criteria eliminated preclinical or *in vitro* research, reviews, meta-analyses, conference abstracts, editorials, and studies lacking methodological detail. Data were extracted and categorized by study design, patient population, UC-MSC source and administration, and clinical outcomes, ensuring a focused dataset by excluding irrelevant studies.

## Results and discussion

Findings from clinical research underscore the significant therapeutic potential of UC-MSCs. With a robust safety profile and the capacity to regenerate tissue and modulate immune responses, UC-MSCs emerge as a promising treatment option for a variety of conditions, including musculoskeletal disorders and immune-related diseases. However, clinical outcomes are inconsistent, influenced by factors such as the specific disease under investigation, the design of clinical trials, and individual patient characteristics. This variability underscores the necessity for standardized methodologies and larger multicenter studies to validate and optimize the application of UC-MSCs. To provide context for this discussion, Table 1 summarizes the primary therapeutic areas explored, the reported benefits, and the current limitations.

**Table 1:** Summary of UC-MSC clinical applications across disease areas

**Title of the study**	**Year**	**Category**	**References**
A Phase I Dose-Escalation Clinical Trial to Assess the Safety and Efficacy of Umbilical Cord-Derived Mesenchymal Stromal Cells in Knee Osteoarthritis. Stem Cells Translational Medicine.	2024	Musculoskeletal disorders	Matas et al. [15]
Umbilical cord-derived mesenchymal stem cells for treating osteoarthritis of the knee: A single-arm, open-label study.	2020	Musculoskeletal disorders	Dilogo et al. [16]
Efficacy of umbilical cord-derived mesenchymal stem cell-based therapy for osteonecrosis of the femoral head: A three-year follow-up study.	2016	Musculoskeletal disorders	Chen et al. [17]
Functional outcome and histologic analysis of late onset total type brachial plexus injury treated with intercostal nerve transfer to median nerve with local umbilical cord-derived mesenchymal stem cells or secretome injection: A double-blinded, randomized control study.	2024	Musculoskeletal disorders	Widodo et al. [18]
A Pilot Study of Mesenchymal Stem Cell Therapy for Acute Liver Allograft Rejection.	2017	Liver diseases	Shi et al. [19]
Mesenchymal stem cell therapy in decompensated liver cirrhosis: A long-term follow-up analysis of the randomized controlled clinical trial.	2021	Liver diseases	Shi et al. [20]
Therapeutic potentials of umbilical cord-derived mesenchymal stromal cells for ischemic-type biliary lesions following liver transplantation.	2017	Liver diseases	Zhang et al. [21]
Allogeneic mesenchymal stem cell as induction therapy to prevent both delayed graft function and acute rejection in deceased donor renal transplantation: Study protocol for a randomized controlled trial.	2017	Kidney diseases/transplantation	Sun et al. [22]
Allogeneic mesenchymal stem cells as induction therapy are safe and feasible in renal allografts: Pilot results of a multicenter randomized controlled trial.	2018	Kidney diseases/transplantation	Sun et al. [23]
Predictive factors that influence the clinical efficacy of umbilical cord–derived mesenchymal stromal cells in the treatment of type 2 diabetes mellitus.	2024	Metabolic/endocrine disorders	Wang et al. [24]
Effects of Maternal Exercise Modes on Glucose and Lipid Metabolism in Offspring Stem Cells.	2023	Metabolic/endocrine disorders	Jevtovic et al. [25]
Umbilical cord-derived mesenchymal stromal cells: Predictive obstetric factors for cell proliferation and chondrogenic differentiation.	2017	Obstetrics/perinatal biology	Avercenc-Léger et al. [26]
Allogeneic mesenchymal stem cells may be a viable treatment modality in cerebral palsy.	2024	Neurological disorders	Boyalı et al. [27]
Therapeutic evidence of umbilical cord-derived mesenchymal stem cell transplantation for cerebral palsy: A randomized, controlled trial.	2020	Neurological disorders	Gu et al. [28]
Effect of umbilical cord mesenchymal stromal cells on motor functions of identical twins with cerebral palsy: Pilot study on the correlation of efficacy and hereditary factors.	2015	Neurological disorders	Wang et al. [29]
Safety and potential efficacy of expanded mesenchymal stromal cells of bone marrow and umbilical cord origins in patients with chronic spinal cord injuries: A phase I/II study.	2024	Neurological disorders	Awidi et al. [30]
Clinical effects of intrathecal administration of expanded Wharton jelly mesenchymal stromal cells in patients with chronic complete spinal cord injury: A randomized controlled study.	2021	Neurological disorders	Albu et al. [31]
Treatment of Optic Canal Decompression Combined with Umbilical Cord Mesenchymal Stem (Stromal) Cells for Indirect Traumatic Optic Neuropathy: A Phase 1 Clinical Trial.	2021	Neurological disorders	Li et al. [32]
Treatment of COVID-19-associated ARDS with umbilical cord-derived mesenchymal stromal cells in the STROMA-CoV-2 multicenter randomized double-blind trial: Long-term safety, respiratory function, and quality of life.	2024	Respiratory disorders/COVID-19	Sitbon et al. [33]
Effect of human umbilical cord-derived mesenchymal stem cells on lung damage in severe COVID-19 patients: A randomized, double-blind, placebo-controlled phase 2 trial.	2021	Respiratory disorders/COVID-19	Shi et al. [34]
Human mesenchymal stem cells treatment for severe COVID-19: 1-year follow-up results of a randomized, double-blind, placebo-controlled trial.	2022	Respiratory disorders/COVID-19	Shi et al. [35]
Human umbilical cord-derived mesenchymal stem cell therapy in patients with COVID-19: A phase 1 clinical trial.	2020	Respiratory disorders/COVID-19	Meng et al. [36]
Mesenchymal stromal cell therapy for COVID-19-induced ARDS patients: a successful phase 1, control-placebo group, clinical trial.	2022	Respiratory disorders/COVID-19	Kaffash Farkhad et al. [37]
Umbilical Cord-derived Mesenchymal Stem Cells modulate TNF and soluble TNF Receptor 2 (sTNFR2) in COVID-19 ARDS patients.	2021	Respiratory disorders/COVID-19	Kouroupis et al. [38]
Treatment of COVID-19-associated ARDS with umbilical cord-derived mesenchymal stromal cells in the STROMA-CoV-2 multicenter randomized double-blind trial: Long-term safety, respiratory function, and quality of life.	2024	Respiratory disorders/COVID-19	Sitbon et al. [33]
Human umbilical cord-derived mesenchymal stromal cells for the treatment of steroid refractory grades III-IV acute graft-versus-host disease with long-term follow-up.	2024	Hematology/immune disorders	Niu J wen et al. [39]
Phase II Multicenter, Randomized, Double-Blind Controlled Study of Efficacy and Safety of Umbilical Cord–Derived Mesenchymal Stromal Cells in the Prophylaxis of Chronic Graft-Versus-Host Disease After HLA-Haploidentical Stem-Cell Transplantation.	2016	Hematology/immune disorders	Gao et al. [40]
Immunological influence of serum-free manufactured umbilical cord-derived mesenchymal stromal cells for steroid-resistant acute graft-versus-host disease.	2022	Hematology/immune disorders	Nagamura-Inoue et al. [41]
Efficacy and safety of human umbilical cord-derived mesenchymal stem cells in the treatment of refractory immune thrombocytopenia: A prospective, single arm, phase I trial.	2024	Hematology/immune disorders	Chen et al. [42]
Safety, immunological effects and clinical response in a phase I trial of umbilical cord mesenchymal stromal cells in patients with treatment refractory SLE.	2022	Hematology/immune disorders	Kamen et al. [43]
*In vitro* Immunomodulatory Effects of Human Umbilical Cord-Derived Mesenchymal Stem Cells on Peripheral Blood Cells from Warm Autoimmune Hemolytic Anemia Patients.	2022	Hematology/immune disorders	Ma et al. [44]
Efficacy and safety of allogeneic umbilical cord-derived mesenchymal stem cells for the treatment of complex perianal fistula in Crohn’s disease: a pilot study.	2023	Gastrointestinal disorders	Wei et al. [45]
Feasibility of reduced-dose posttransplant cyclophosphamide and cotransplantation of peripheral blood stem cells and umbilical cord-derived mesenchymal stem cells for SAA.	2021	Hematopoietic disorders	Zu et al. [46]

Abbreviations: ARDS: Acute respiratory distress syndrome; TNF: Tumor necrosis factor; sTNFR2: Soluble tumor necrosis factor receptor 2; HLA: Human leukocyte antigen; SLE: Systemic lupus erythematosus; SAA: Severe aplastic anemia; STROMA-CoV-2: Multicenter randomized double-blind trial acronym in COVID-19-associated ARDS.

### UC-MSCs in musculoskeletal regeneration

Clinical studies increasingly demonstrate that UC-MSCs hold significant potential for treating musculoskeletal disorders, although the strength of evidence varies across trials. In a phase I dose-escalation study, Matas et al. [15] found that intra-articular UC-MSC injections in knee osteoarthritis were safe and resulted in improvements in pain and joint function, providing early proof of concept for their application in degenerative joint diseases. These findings align with those of Dilogo et al. [16], whose open-label study reported similar benefits, further validating the feasibility of UC-MSC therapy in osteoarthritis. Expanding the evidence base, Chen et al. [17] presented three-year follow-up data in patients with osteonecrosis of the femoral head, showing not only symptom relief but also a deceleration of disease progression, suggesting a more durable regenerative effect. Beyond joint diseases, Widodo et al. [18] investigated UC-MSCs in late-onset brachial plexus injury, demonstrating that either the cells or their secretome enhanced functional recovery and tissue repair, indicating a broader role in complex neuromuscular injuries. Collectively, these studies underscore the safety, regenerative potential, and versatility of UC-MSCs in musculoskeletal medicine. However, comparisons among studies are complicated by variations in trial design, patient populations, and treatment protocols. Improvements in osteoarthritis outcomes are consistent across short-term studies, while the long-term benefits observed in osteonecrosis necessitate further confirmation. The findings concerning brachial plexus injuries are promising but remain preliminary and require validation in larger trials. Overall, the evidence suggests that UC-MSCs could revolutionize treatment for musculoskeletal disorders, provided that future multicenter studies optimize dosing strategies, standardize methods, and confirm both safety and long-term efficacy.

### UC-MSCs in liver disease

Clinical research into UC-MSCs in liver disease indicates a broad therapeutic potential across conditions such as cirrhosis, transplantation, and biliary complications. In a long-term follow-up study, Shi et al. [19] demonstrated that UC-MSC therapy in patients with decompensated cirrhosis led to sustained improvements in liver function scores and overall clinical status, highlighting both the regenerative and immunomodulatory effects of the therapy. These findings provide critical long-term evidence that UC-MSCs can stabilize chronic liver disease, a condition with limited effective treatment options outside of transplantation. Complementing this, Shi et al. [20] reported in a pilot trial that UC-MSC infusion was safe and clinically beneficial for patients experiencing acute liver allograft rejection. Here, MSCs not only reduced inflammatory activity but also appeared to support graft survival, suggesting that their immunoregulatory properties may be harnessed in transplant settings where rejection remains a significant challenge. Extending to biliary complications, Zhang et al. [21] explored UC-MSCs in patients with ischemic-type biliary lesions following transplantation. Their findings indicated improved biliary repair and function, with the therapy being well tolerated. Together, these studies illustrate that UC-MSCs operate through a combination of regenerative and immunomodulatory mechanisms to enhance outcomes across diverse liver-related conditions. While the long-term cirrhosis trial underscores their potential in managing chronic diseases, the transplant-focused studies highlight their role in addressing acute immune-mediated injury and post-transplant complications. Nevertheless, the evidence base remains relatively small, predominantly consisting of pilot studies and single-center trials. Larger, multicenter randomized studies are essential to confirm efficacy, standardize treatment protocols, and determine how UC-MSC therapy can be integrated into existing liver disease and transplantation frameworks. Current data, however, suggest that UC-MSCs are a versatile tool capable of addressing both chronic degeneration and acute immune-driven injury in hepatology.

### UC-MSCs in renal transplantation

Research on UC-MSCs in renal transplantation has primarily focused on their potential as induction therapy to enhance graft survival and mitigate immune complications. Sun et al. [22] first outlined a comprehensive study protocol investigating whether allogeneic MSCs could prevent delayed graft function and acute rejection in recipients of deceased donor kidneys. This protocol established a scientific rationale for introducing MSCs at the time of transplantation, aiming to leverage their immunomodulatory and anti-inflammatory properties during the critical early post-transplant phase. Building on this framework, Sun et al. [23] later published pilot results from a multicenter randomized controlled trial, demonstrating that UC-MSC induction therapy was both safe and feasible in renal allografts. Importantly, early findings indicated improvements in graft function and reduced immune-mediated injury compared to standard care. Collectively, these studies illustrate a methodical progression from conceptual design to clinical application. The protocol paper laid the groundwork for methodological rigor, while the subsequent clinical trial provided preliminary evidence that UC-MSCs may enhance short-term transplant outcomes without presenting significant safety concerns. However, both studies remain early in scope, with small sample sizes and short follow-up limiting conclusions about long-term efficacy. Larger, multicenter trials with extended monitoring will be critical to determine whether UC-MSC therapy can reduce rejection rates, improve long-term graft survival, and ultimately be incorporated into standard transplantation protocols.

### Metabolic applications of UC-MSCs

Research into the metabolic applications of UC-MSCs reveals both their direct therapeutic potential and the influence of maternal and obstetric factors on their biological properties. In patients with type 2 diabetes mellitus, Wang et al. [24] identified predictive factors that influenced the clinical efficacy of UC-MSC therapy, demonstrating that treatment improved glucose control, insulin sensitivity, and systemic inflammation in responders. This study emphasizes that patient-specific characteristics may shape therapeutic outcomes, highlighting the importance of precision in applying UC-MSC therapy for metabolic disorders. Concurrently, maternal factors have been shown to affect the biological function of UC-MSCs. Jevtovic et al. [25] demonstrated that maternal exercise during pregnancy positively influenced glucose and lipid metabolism in offspring stem cells, suggesting that lifestyle factors can program neonatal cell biology in ways that may enhance their regenerative and metabolic potential. Similarly, Avercenc-Léger et al. [26] reported that certain obstetric conditions predicted UC-MSC proliferation and chondrogenic differentiation capacity, providing further evidence that the perinatal environment directly impacts stem cell quality and function. Together, these studies expand the understanding of UC-MSCs beyond their therapeutic effects in established diseases to include the maternal and perinatal factors that shape their baseline biology.

### UC-MSCs for neurological disorders

Clinical investigations into UC-MSCs for neurological disorders highlight their potential to promote functional recovery, although findings vary depending on the condition and study design. In cerebral palsy, Boyalı et al. [27] provided preliminary evidence that allogeneic MSC therapy may be a viable treatment, reporting improvements in motor function with good tolerability. These results were reinforced by Gu et al. [28], who conducted a randomized controlled trial showing significant gains in gross motor function compared with controls, underscoring the therapeutic promise of UC-MSCs in pediatric neurorehabilitation. Adding nuance, Wang et al. [29] studied identical twins with cerebral palsy and observed improvements in motor function, while also suggesting that hereditary factors may influence the degree of clinical response, an important consideration for tailoring treatment strategies. Spinal cord injury has also been a major focus of UC-MSC research. Awidi et al. [30] demonstrated in a phase I/II trial that expanded stromal cells from both bone marrow and umbilical cord were safe and feasible in chronic spinal cord injury, with some patients experiencing neurological improvement. Similarly, Albu et al. [31] investigated intrathecal administration of Wharton’s jelly-derived MSCs and reported improvements in sensory and motor recovery, further affirming both safety and therapeutic potential in this challenging patient population. Extending the scope to neuro-ophthalmology, Li et al. [32] combined optic canal decompression with UC-MSC transplantation for traumatic optic neuropathy. Their phase I results showed that the procedure was safe and provided preliminary indications of visual function improvement. Collectively, these studies suggest that UC-MSCs exert neuroprotective and regenerative effects across a range of neurological conditions, from developmental disorders such as cerebral palsy to traumatic injuries of the spinal cord and optic nerve. While improvements in motor and sensory outcomes appear consistent, the magnitude of benefit varies, and long-term durability remains to be fully established. Variability in cell sources, administration routes, and patient populations further complicates direct comparisons. Nonetheless, the convergence of evidence across multiple indications highlights UC-MSCs as a versatile therapeutic platform for neuroregeneration. Future research should focus on larger randomized trials, standardized treatment protocols, and long-term follow-up to clarify the extent and durability of neurological recovery achievable with UC-MSC therapy.

### UC-MSCs for respiratory disorders

Clinical research into UC-MSCs for respiratory disorders, particularly acute respiratory distress syndrome (ARDS) associated with COVID-19, has demonstrated promising safety and early efficacy signals, although results vary across trials. In a multicenter randomized double-blind trial, Sitbon et al. [33] reported that UC-MSC therapy was safe and improved respiratory function and quality of life in specific patient subsets, suggesting meaningful long-term benefits. Similarly, Shi et al. [34] confirmed sustained improvements in pulmonary outcomes in their one-year follow-up study of severe COVID-19 patients, reinforcing the therapy’s durability and safety profile. Earlier work by Shi et al. [35] indicated that UC-MSC treatment reduced lung damage and improved radiological outcomes, providing mechanistic evidence of tissue repair. In a phase I trial, Meng et al. [36] reported that UC-MSC infusions were well tolerated and associated with signs of clinical improvement. Additionally, Kaffash Farkhad et al. [37] observed improvements in oxygenation and disease severity in a controlled phase I study, further supporting the feasibility of UC-MSC use in acute respiratory injury. Complementing these clinical outcomes, Kouroupis et al. [38] explored immunological mechanisms, demonstrating that UC-MSCs modulated TNF and soluble TNF receptor 2 levels in patients with COVID-19 ARDS, thereby supporting the hypothesis that their therapeutic benefits are mediated through immunomodulation and dampening of hyperinflammatory pathways. Collectively, these studies highlight several consistent themes: UC-MSCs are safe across diverse patient populations, improve short-term oxygenation and radiological findings, and may contribute to longer-term recovery of lung function. However, trial heterogeneity—including differences in sample size, dosing regimens, and outcome measures—limits definitive conclusions. While phase I and pilot studies provide encouraging feasibility data, larger multicenter randomized trials, such as those led by Sitbon et al. [33], are critical for validating efficacy and informing clinical guidelines. Overall, UC-MSCs appear to offer dual benefits in ARDS by reducing inflammation and promoting lung repair, yet robust evidence from large-scale studies is still required before their integration into standard respiratory care.

### UC-MSCs in immune-mediated and transplant-related disorders

Clinical studies investigating UC-MSCs in immune-mediated and transplant-related disorders demonstrate consistent safety and notable immunomodulatory benefits, though variability in outcomes is observed depending on the disease context. In graft-versus-host disease (GVHD), Niu et al. [39] reported durable clinical responses in patients with severe, steroid-refractory GVHD, with long-term follow-up confirming sustained improvements and manageable safety concerns. Expanding on this, Gao et al. [40] conducted a multicenter randomized controlled trial and found that prophylactic use of UC-MSCs significantly reduced the incidence and severity of chronic GVHD, highlighting their potential role in both prevention and treatment. Similarly, Nagamura-Inoue et al. [41] provided mechanistic insight, demonstrating that serum-free manufactured UC-MSCs shifted immunological responses in steroid-resistant GVHD without increasing infection risk, underscoring their capacity to restore immune balance safely. Beyond transplantation, UC-MSCs have been tested in autoimmune and inflammatory diseases. Chen et al. [42] showed that UC-MSC infusion in refractory immune thrombocytopenia improved platelet counts and reduced bleeding events in a subset of patients, while Kamen et al. [43] reported reductions in disease activity indices in systemic lupus erythematosus, confirming both safety and immunological benefit. Complementing these clinical findings, Ma et al. [44] provided *in vitro* evidence that UC-MSCs suppressed pathogenic immune activity in autoimmune hemolytic anemia, reinforcing the rationale for their use in autoimmune conditions. In inflammatory bowel disease, Wei et al. [45] reported higher closure rates of complex perianal fistulas in Crohn’s disease patients treated with UC-MSCs, demonstrating benefits in tissue repair alongside immune regulation. UC-MSCs have also been explored as supportive therapy in hematologic transplantation. Zu et al. [46] combined UC-MSCs with reduced-dose cyclophosphamide and peripheral blood stem cells in patients with severe aplastic anemia, reporting encouraging engraftment and improved GVHD control with acceptable toxicity. This study highlights how UC-MSCs function synergistically in cotransplant settings to enhance outcomes. Collectively, these studies consistently underscore the ability of UC-MSCs to modulate immune responses across diverse conditions, from GVHD and autoimmune cytopenias to Crohn’s disease and systemic lupus erythematosus, while maintaining a strong safety profile.

### Critical barriers to the clinical integration of UC-MSCs

Although the accumulated evidence highlights the therapeutic promise of UC-MSCs, several important limitations must be acknowledged before their clinical use can be fully established. Findings across studies are not always consistent. For instance, clinical trials in neurological disorders, such as cerebral palsy and spinal cord injury, have reported variable outcomes: some demonstrated substantial gains in motor function and quality of life, while others noted only modest or short-lived improvements. Such discrepancies are likely influenced by differences in cell origin (e.g., Wharton’s jelly vs cord blood), methods of expansion and preparation, delivery routes (intravenous, intrathecal, or scaffold-based), and heterogeneity in patient characteristics, such as age, disease stage, and comorbidities. Likewise, trials in liver and renal disorders have shown promising but uneven results, underscoring the importance of developing standardized protocols for cell preparation, dosing, and administration. Without harmonization, direct comparisons between studies remain challenging, and conclusions about efficacy are tentative [47].

Another critical limitation of the current literature is its reliance on small, early-phase clinical studies. Most trials enroll fewer than 50 patients and follow them for less than a year. While these studies demonstrate safety and short-term efficacy, they cannot provide definitive evidence of long-term outcomes, including durability of therapeutic benefit or the risk of late adverse effects such as unwanted immune reactions, fibrosis, or tumorigenicity. Moreover, inconsistencies in reporting methods and outcome measures hinder meaningful meta-analyses and systematic reviews, which are essential for translating preclinical and early clinical findings into widely accepted treatment guidelines. Currently, the absence of uniform standards in trial design, patient selection, and clinical endpoints remains a significant obstacle to progress [48]. Additionally, several translational and regulatory barriers must be addressed before UC-MSCs can transition from experimental therapy to mainstream clinical practice. Large-scale production under good manufacturing practice (GMP) conditions necessitates rigorous quality control to ensure product consistency, viability, and potency. The regulatory landscape is fragmented, with differing approval processes and safety requirements across regions, complicating global commercialization. Logistical challenges related to biobanking, cryopreservation, transport, and cost-effectiveness must also be resolved for UC-MSC therapy to be scalable and accessible. Addressing these issues will require coordinated international efforts, multicenter randomized controlled trials with long-term follow-up, and continued innovation in cell engineering and delivery strategies. Only then can UC-MSCs progress from promising experimental interventions to standardized, evidence-based therapies that transform routine clinical care [49].

## Conclusion

UC-MSCs represent a significant advancement in regenerative medicine, demonstrating potential across a diverse array of medical conditions. Current evidence indicates that UC-MSCs can facilitate immune regulation, tissue repair, and recovery in both chronic and acute diseases, such as autoimmune disorders, liver cirrhosis, neurological injuries, chronic kidney disease, osteoarthritis, and COVID-19-related ARDS. Their unique biological properties—such as a low risk of immune rejection, robust paracrine signaling, and high proliferative capacity—render them a valuable therapeutic option for patients whose needs are inadequately addressed by existing treatments.

However, the field remains nascent. Much of the existing data is derived from small pilot or Phase I/II studies with limited follow-up periods, making it challenging to comprehensively evaluate the long-term safety and durability of treatment effects. Moreover, variability in the sourcing, preparation, and administration of UC-MSCs, as well as differences in patient demographics, contribute to inconsistencies that hinder direct comparisons across clinical trials.

In addition to scientific challenges, obstacles such as large-scale manufacturing, high costs, and divergent international regulatory frameworks further impede the clinical adoption of UC-MSC therapies. To transition from experimental applications to standard medical practice, large multicenter randomized controlled trials utilizing standardized protocols and extended follow-up are imperative. Furthermore, achieving greater alignment in global regulatory standards and enhancing manufacturing processes will be crucial.

Looking ahead, integrating UC-MSC therapy with innovations in biomaterials, gene editing, or exosome-based strategies may unlock even greater therapeutic potential. With continued advancements, UC-MSCs have the capacity to evolve from promising experimental treatments to foundational elements of future regenerative and personalized medicine, providing enduring benefits for conditions that are among the most challenging to treat.
